# A novel feature of the ancient organ: A possible involvement of the subcommissural organ in neurogenic/gliogenic potential in the adult brain

**DOI:** 10.3389/fnins.2023.1141913

**Published:** 2023-03-07

**Authors:** Hitoshi Inada, Laarni Grace Corales, Noriko Osumi

**Affiliations:** ^1^Laboratory of Health and Sports Sciences, Division of Biomedical Engineering for Health and Welfare, Graduate School of Biomedical Engineering, Tohoku University, Sendai, Japan; ^2^Department of Developmental Neuroscience, Graduate School of Medicine, Tohoku University, Sendai, Japan

**Keywords:** subcommissural organ, neurogenesis, neural stem progenitor cell, Pax6, Sox2

## Abstract

The subcommissural organ (SCO) is a circumventricular organ highly conserved in vertebrates from *Cyclostomata* such as lamprey to mammals including human. The SCO locates in the boundary between the third ventricle and the entrance of the aqueduct of Sylvius. The SCO functions as a secretory organ producing a variety of proteins such as SCO-spondin, transthyretin, and basic fibroblast growth factor (FGF) into the cerebrospinal fluid (CSF). A significant contribution of the SCO has been thought to maintain the homeostasis of CSF dynamics. However, evidence has shown a possible role of SCO on neurogenesis in the adult brain. This review highlights specific features of the SCO related to adult neurogenesis, suggested by the progress of understanding SCO functions. We begin with a brief history of the SCO discovery and continue to structural features, gene expression, and a possible role in adult neurogenesis suggested by the SCO transplant experiment.

## Introduction

**FIGURE 1:**
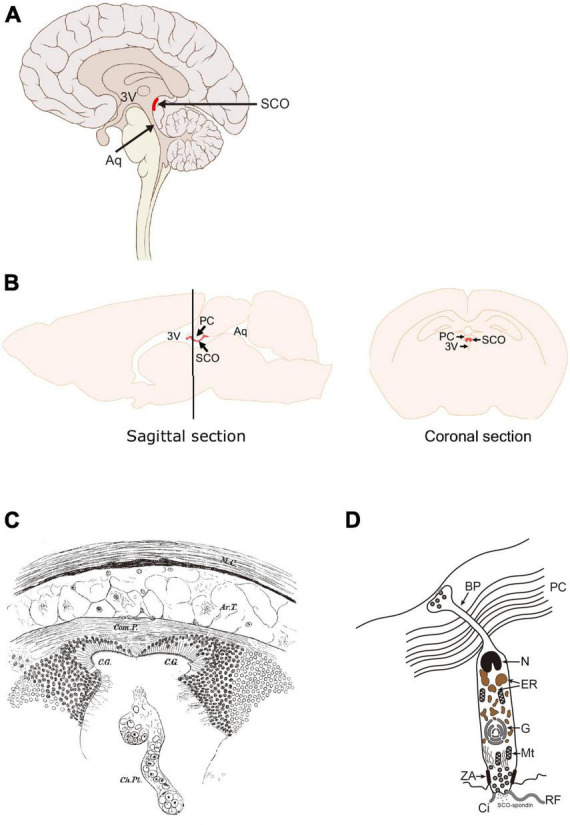
Location of the SCO in the adult human and rodent brains. **(A)** Sagittal section of human brain. **(B)** Sagittal and coronal sections of rodent brains. The SCO locates at the boundary between the third ventricle (3V) and the entrance of the aqueduct of Sylvius (Aq). The SCO is composed of an ependymal cell layer(s) lining the third ventricular side of the posterior commissure. In the coronal section, the SCO appears as an inverted U-shape underneath the posterior commissure (PC). 3V, third ventricle; Aq, aqueduct of Sylvius; PC, posterior commissure; SCO, subcommissural organ (Corales et al., 2022). **(C)** Illustration of “a pair of ciliated grooves”. Ar.T., arachnoidal tissue; C.G., ciliated groove; Ch.PL, choroid plexus; Com.P., posterior commissure; M.C., connective tissue brain case. Used with permission of The Royal Society (U.K.), from Dendy (1902); permission conveyed through Copyright Clearance Center, Inc. **(D)** The secretory feature of the SCO ependymal cell. Scheme of an SCO secretory ependymal cell. The secretory proteins such as SCO-spondin and transthyretin are stored in the endoplasmic reticulum (ER), modified in the Golgi apparatus (G), and released both apically into the CSF and basally into the matrix of the posterior commissure. The SCO-spondin released into the CSF forms the RF. BP, basal process; Ci, cilia; ER, endoplasmic reticulum; G, Golgi apparatus; Mt, mitochondria; N, nucleus; PC, posterior commissure; RF, Reisner’s fiber; ZA, zonula adherens.

The subcommissural organ (SCO) is a circumventricular organ with a long history but is still enigmatic (Oksche et al., 1993; Meiniel et al., 1996; Rodriguez and Yulis, 2001; Kiecker, 2018). From *Cyclostomata* such as lamprey to mammals, the SCO is substantially conserved among vertebrates. The SCO is located at the boundary between the third ventricle and the entrance to the aqueduct of Sylvius (Figures 1A, B; Duvernoy and Risold, 2007; Kiecker, 2018; Corales et al., 2022). Its importance to maintaining the homeostasis of cerebrospinal fluid (CSF) dynamics has drawn attention to its functions (Perez-Figares et al., 2001; Guerra et al., 2015). The SCO has been hypothesized to play a role in neurogenesis (Guerra et al., 2015), although extensive research on dynamics of CSF and neuropathology of hydrocephalus has been conducted. Recent advancement in our understanding of the function of the SCO have led us to hypothesize that the SCO possesses features relevant to adult neurogenesis. In this review, we overview how the SCO is discovered before discussing its structural characteristics, gene expression, and discuss a potential involvement in adult neurogenesis suggested by the SCO transplant experiment.

## A brief history of the SCO

**FIGURE 2:**
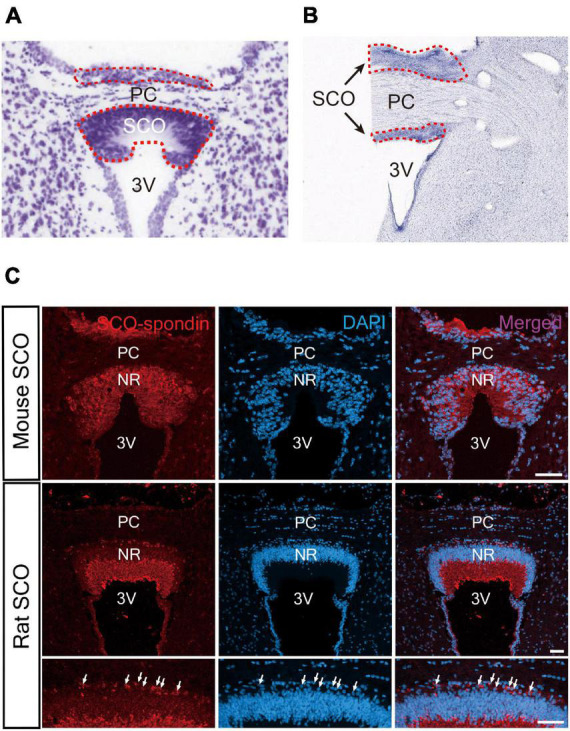
Histological structure of the SCO. **(A)** The SCO region of mouse brain. The SCO is composed of an ependymal cell layer(s) with an inverted U-shape lining the third ventricular side of the posterior commissure (Allen Brain Atlas: Mouse Brain, https://atlas.brain-map.org/). **(B)** The SCO region of human brain (BrainSpan Atlas of the Developing Human Brain, https://atlas.brain-map.org/). The SCO regions are surrounded by red dotted lines. **(C)** Immunostaining of SCO-spondin in the SCO region of the adult mouse and rat brain. The SCO-spondin staining pattern in the adult rat SCO shows a weak staining pattern in the nuclear region compared to the adult mouse SCO allowing better visualization of hypendymal cells between the ependymal layer and the posterior commissure. Arrows indicate hypendymal cells. Scale bars: 50 μm (Corales et al., 2022). 3V, third ventricle; Ep, ependymal cells; NR, nuclear region; PC, posterior commissure.

The first clear description of the SCO appears in the 1900’s (Dendy, 1902). In an anatomical study on ammocoetes, a New Zealand Lamprey (*Geotria australis*), the SCO was described as “*a pair of ciliated grooves*” (Figure 1C), which in earlier study was referred as the epithelial layer (Edinger, 1892; Studnicka, 1900). The epithelium of the SCO is distinct from those in other brain regions due to its cylindrical structure. Dendy described it as follows; “They are most conspicuous beneath the commissure itself (figs. 1, 2), in which region they are lined by a sharply defined epithelium of very long columnar cells, totally different in appearance from the epithelium which lines the remainder of the brain-cavity.” (Dendy, 1902). The SCO function was speculated, at that time, as making the circulation of the brain fluid due to its ciliated form and location. The term “Sub-Commissural Organ” was first proposed by Dendy and Nicholls in Dendy and Nicholls (1910). The same report also mentioned that the SCO exists in higher vertebrates such as mice, cats, and chimpanzees. At this time, “a pair of ciliated grooves” or “the epithelial layer beneath posterior commissure” was established as the “Sub-Commissural Organ.”

## Structural feature of the SCO

The SCO surface is sparsely ciliated and covered with microvilli, contrasting with the other ependymal areas, which are composed with multiciliated cells (Collins and Woollam, 1979; Rodriguez et al., 1998). The SCO consists of an inverted U-shape ependymal layer(s) lining the third ventricular side of the posterior commissure in the coronal section (Collins and Woollam, 1979; Rodriguez et al., 1998; Figures 2A, B). Later, it was proposed that the SCO is composed of two layers, ependyma and hypendyma, in vertebrates (Rodriguez et al., 1984). A small number of hypendymal cells are observed in amphibia and reptiles such as frogs, lizards, and snakes. In contrast, the hypendymal layer is more distinct in larger mammals such as bovines and primates, but its existence is species-dependent (Rodriguez et al., 1984). It appears that the presence of hypendymal cells is species-specific and be uncertain in animals with smaller brains. For example, a hypendymal layer is observed in rats but not in mice (Figure 2C; Corales et al., 2022).

As stated in the previous section, the SCO has been considered a secretory organ due to its cylindrical structure (Figure 1D). The SCO cells have an elongated shape, compared to typical ependymal cells, a large nucleus at the basal side adjacent to the posterior commissure (PC), the endoplasmic reticulum and the Golgi apparatus containing secretary molecules such as SCO-spondin and transthyretin (TTR). The apical pole of the SCO cells is exposed to the ventricular cavity. Apparent zonulae adherence is observed to connect adjacent cells. SCO-spondin secreted from the apical side forms the Reisner’s fiber (RF) (Meiniel, 2001). This unique structure is also confirmed by a study using electron microscopy in detail (Meiniel, 2007).

## Gene expression related to brain development in the SCO cells

Our knowledge on molecules expressed in the SCO is limited because of its restricted region, although a study reported a systematic DNA-chip analysis of circumventricular organs including SCO (Szathmari et al., 2013).

### Secretory proteins

Due to their feature as secretory cells, two major secretory proteins, SCO-spondin and TTR, have been well studied in the SCO (Gobron et al., 1996; Montecinos et al., 2005).

**FIGURE 3:**
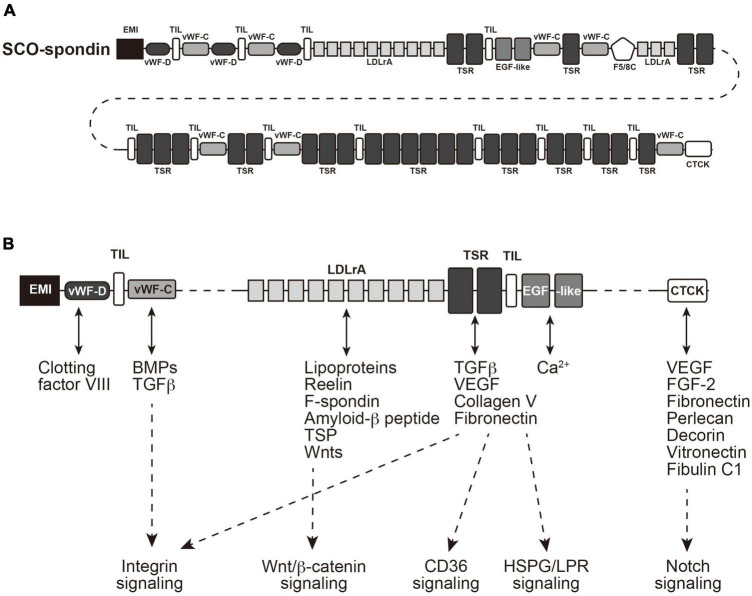
Structure of SCO-spondin and its downstream signaling pathways. **(A)** A cartoon of SCO-spondin structure. EMI, elastin microfibril interface; vWF-D, von Willebrand factor type-D; FA5/8C, Factor V/Factor VIII type C; LDLrA, low-density lipoprotein receptor class A; TIL, trypsin inhibitor-like; TSR thrombospondin type I repeat; vWF-C, von Willebrand factor type-C; EGF, epidermal growth factor; CTCK, C-terminal cystine knot-like. **(B)** Possible interactions of domains contained in SCO-spondin with soluble factors. FGF2, fibroblast growth factor 2; TGF-β, transforming growth factor-β; VEGF, vascular endothelial growth factor; HSPG, heparan sulfate proteoglycan; LPR, LDLr-related protein.

Subcommissural organ-spondin is a giant glycoprotein with a large molecular weight (∼540 kDa), which has been isolated as the SCO-specific transcript and identified as a component of Reissner’s fiber (Meiniel et al., 1995; Gobron et al., 1996; Creveaux et al., 1998; Meiniel, 2001). SCO-spondin contains many functional domains (Figure 3A); elastin microfibril interface (EMI) domain, von Willebrand factor type-D (vWF-D) domains, FA5/8C domain, Low-density lipoprotein receptor class A (LDLrA) domains, trypsin inhibitor-like (TIL) domains, thrombospondin type I repeat (TSR) domains, von Willebrand factor type-C (vWF-C) domains, EGF-like domains, and CTCK domain (Gobron et al., 2000; Meiniel and Meiniel, 2007; Aboitiz and Montiel, 2021; Sepulveda et al., 2021). The SCO-spondin and its degradation products containing these domains have been proposed to function as neurogenesis regulators (Vera et al., 2013, 2015). SCO-spondin is also reported to have a neuroprotective effect (Deletage et al., 2021). Since the SCO-spondin contains many domains interacting with soluble factors, such as fibroblast growth factor 2 (FGF2), transforming growth factor-β (TGF-β), and vascular endothelial growth factor (VEGF), derivatives of the SCO-spondin could function as carriers or chelators of those soluble factors to regulate downstream signaling pathways (Figure 3B).

Transthyretin is a carrier for thyroid hormones in CSF (Alshehri et al., 2015; Richardson et al., 2015). It has previously been considered that only the choroid plexus produces TTR in the brain, although the SCO is shown to secrete TTR into the CSF (Montecinos et al., 2005). TTR might contribute to adult neurogenesis by regulating thyroid hormone homeostasis (Kapoor et al., 2015) since adult neural stem cell cycling *in vivo* requires thyroid hormone and its alpha receptor (Lemkine et al., 2005). Cell division and apoptosis are affected at the neural stem cell niche in the TTR null mice (Richardson et al., 2007).

The adult rat SCO shows strong expression of FGF2 (also known as a basic fibroblast growth factor, b-FGF) (Cuevas et al., 1996). FGF2 is critical in maintaining adult neurogenesis in the neurogenic niches (Mudo et al., 2009; Woodbury and Ikezu, 2014). The SCO also expresses Wnt1, a secretory glycoprotein critical for morphogenesis during brain development. Mutation in Wnt1 caused abnormal differentiation of the SCO in the mouse embryo (Louvi and Wassef, 2000). Wnt1 expression is observed in the SCO at the RNA level based on Allen Mouse Brain Atlas.^1^ Wnt signaling pathway has been reported to regulate neural differentiation from neural stem/progenitor cell (NSPCs) in early embryonic brain development (Hirabayashi et al., 2004; Machon et al., 2007; Munji et al., 2011; Inestrosa and Varela-Nallar, 2015).

These secretory proteins from the SCO could contribute to neurogenesis in the embryonic brain and adult neurogenesis (discussed later). In addition, these secretory proteins could also contribute to maintaining the SCO functions in an autocrine manner since the FGF2 receptor is expressed in the SCO (Szathmari et al., 2013).

The SCO development is consistent during embryonic stages among species, but its maintenance appears species dependent. For example, the SCO begins to differentiate at embryonic day 12.5 (E12.5) in mice, well developed by E16.5 (Estivill-Torrús et al., 2001), and appears to be maintained through postnatal stages (Corales et al., 2022). On the other hand, the SCO is well developed at embryonic stages (3- to 5-month-old fetuses) but gradually regresses the SCO structure after 5-month-old fetuses (Rodriguez et al., 2001). The SCO cells are significantly reduced in 1-year-old infants and lose their secretory features in the adult stages (Rodriguez et al., 2001).

### Transcription factors

It has been reported that several transcription factors are critical for the formation of the SCO. Ectopic expression of Engrailed 1 (En1), a homeobox transcription factor related to Wnt1 signaling, interferes with the differentiation of circumventricular organs, including the SCO (Louvi and Wassef, 2000). En1 expression is also confirmed in the SCO at the RNA level based on Allen Brain Atlas: Mouse Brain.^2^ Deficiency in the function of Pax6, an essential transcription factor for neurogenesis in the embryonic and adult brain (Osumi et al., 2008), results in impairment of the SCO differentiation in early brain development (Estivill-Torrús et al., 2001). The SCO’s structural and functional features as a secretory organ are completely lacking in the *Pax6^Sey/Sey^* mutant mouse, suggesting that Pax6 is critical for SCO formation. Msx1, a homeodomain transcription factor, is expressed in the SCO of the mouse embryonic brain, and a mutation in *Msx1* gene causes a defect in the SCO formation (Bach et al., 2003; Ramos et al., 2004). However, since these transcription factors are related to regionalization in early brain development, it remains unknown whether abnormality in the SCO might be directly caused by defects in these transcription factors or secondarily induced by compartmentation deficits.

Expressions of RFX3 and RFX4, members of the regulatory factor X gene family related to ciliogenesis, are also reported in the SCO. Their mutation or misexpression causes a severe hydrocephalus phenotype in mice, possibly by malformation of the SCO (Blackshear et al., 2003; Baas et al., 2006; Xu et al., 2018). Functional expression of CREB was also confirmed in the isolated SCO cells in an aspect to the cAMP-PKA pathway (Nurnberger and Schoniger, 2001; Schoniger et al., 2002).

As mentioned above, the secretory proteins from SCO could be associated with several signaling pathways, such as integrin, Wnt/β-catenin, and Notch signaling pathways (Figure 3B). Recent studies suggested that these signaling pathways are involved in multiciliated cell differentiation or hydrocephalus (Failler et al., 2021; Lewis and Stracker, 2021; Liu et al., 2023), emphasizing critical SCO roles on ciliogenesis and hydrocephalus caused by its malfunction.

## Proliferation and differentiation potential of SCO cells: Neurogenic/gliogenic and embryonic/adult

**FIGURE 4:**
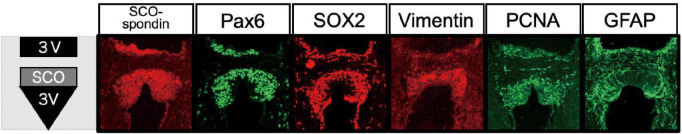
Expression of NSPC markers in the SCO. Immunostaining of NSPC markers in the mouse SCO (Corales et al., 2022).

A significant role of the SCO is maintaining CSF homeostasis by forming RF, which consists of SCO-spondin. However, several lines of evidence suggest its contribution to adult neurogenesis. Recently, we have reported that the SCO cells have a unique feature as immature neuroepithelial cells in the adult mouse brain (Corales et al., 2022). The SCO cells in the adult brain expressed known NSPC markers, i.e., Pax6, Sox2, and vimentin, and a proliferating marker, PCNA (Figure 4). Neither expression of another proliferation marker Ki67, indicating a G2/M phase, nor incorporation of BrdU, an indicator for DNA synthesis in the S phase, are undetectable, suggesting that the SCO cells have a potential for proliferation but are quiescent for cell division in the adult. The SCO cells also express other neuroepithelial cell markers, such as Nestin, as well as Notch 1, Hes1 and Hes3, Occludin, E-cadherin, MSI-1, Sox9, and BMI-1 at the RNA level based on Allen Brain Atlas.^3^ These data demonstrate that the adult SCO cells maintain neuroepithelial cell characteristics, suggesting that the SCO is a possible adult neural stem cell niche.

The quiescent SCO might express its neurogenic activity by appropriate stimuli. For example, tanycytes in the adult hypothalamus are a subtype of ependymal cells with a long radial process (Rodriguez et al., 2005). It has been shown that sub-population of the tanycytes contain stimuli-responsive NSPCs (Lee et al., 2012; Cheng, 2013; Robins et al., 2013; Maggi et al., 2014). A recent study has reported that tanycyte-like ependymal cells in circumventricular organs (CVOs) and the central canal (CC) show a neural stem cell-like phenotype in the adult mouse brain (Furube et al., 2020). In the study, tamoxifen-induced EGFP labeling under the control of *Nestin-CreERT2* transgene has identified NSPCs and shown that the EGFP-labeled ependymal cells distribute in the organum vasculosum laminae terminalis (OVLT), subfornical organ (SFO), CC, and the arcuate nucleus (Arc) of the hypothalamus. Furthermore, EGFP-labeled ependymal cells increased by stimulation with FGF-2/EFG (Furube et al., 2020). The EGFP-labeled tanycyte-like ependymal cells of the OVLT and SFO express both GFAP and Sox2 but not Pax6, while the cells of the CC express those three marker proteins (Furube et al., 2020), which is similar to our results in the SCO (Corales et al., 2022). However, no EGFP-labeled ependymal cells in the SCO were mentioned in the study (Furube et al., 2020). Possibly, the SCO might be activated by the other factors than FGF-2/EFG. In addition, SCO cells are distinct from the tanycytes by structural features. The SCO cells have primary cilia while tanycytes or tanycyte-like cells are unciliated or unciliated/bi-ciliated, respectively (Langlet et al., 2013; Mirzadeh et al., 2017). The cytoplasm of the tanycyte often shows smooth endoplasmic reticulum (Wittkowski, 1998), while the cytoplasm of SCO cells are filled with rough endoplasmic reticulum (Rodriguez et al., 2001), consistent with their secretory function. Therefore, even though the SCO cells and tanycytes share a part of NSPC marker expression, they are different subtypes of ependymal cells.

Although the SCO cells share many properties with NSPCs in the gene expression, it is difficult to clarify whether they could express proliferation activity and whether they are neurogenic RG-like cells and/or gliogenic progenitors. At this point, what kind of cells could be produced from the SCO remains unknown. Considering its location near the posterior commissure and the existence of oligodendrocyte precursor cells (OPCs) at the periphery of the SCO (Corales et al., 2022), the SCO might be a niche for oligogenesis rather than neurogenesis.

Another possible involvement of the SCO in adult neurogenesis is obtained by transplantation of SCO cells into a lateral ventricle. In the study by Rodríguez et al., tissue blocks containing the SCO and the PC were transplanted into the left lateral ventricle in 2–3 months-old Sprague–Dawley rats (Rodriguez et al., 1999). It is observed that the grafted SCOs keep an ultrastructure similar to those of the SCO *in situ* and have the ability of production and secretion of SCO-spondin, resulting in the RF in the explanted lateral ventricle. The transplantation using bovine SCO explants also support that the secretory active SCO can induce cell proliferation. The bovine SCO cultured *in vitro* for a few weeks express and secrete the SCO-spondin and TTR into the culture medium (Schobitz et al., 2001; Montecinos et al., 2005). Xenografts of bovine SCO explants into a lateral ventricle of rats indeed promote cell proliferation in the ipsilateral than contralateral SVZ niche (Guerra et al., 2015), suggesting that the SCO might contribute to the proliferation of NSPCs and possibly leading to neurogenesis through secreted factors CSF circulation in the adult brain.

One possible interpretation for the increased PCNA positive cells by the SCO transplant is that neuroprotective factors secreted by the grafted SCO might enhance or maintain cell proliferation, as suggested in the previous study where the SCO secretes SCO-spondin or TTR (Schobitz et al., 2001; Montecinos et al., 2005). This possibility can be supported by the contribution of the SCO to the early embryonic brain development. Especially, the SCO has been reported to show its contribution to neurogenesis during embryogenesis. In the embryonic stages, the SCO seems to regulate cell proliferation and neuronal differentiation through the secretion of the SCO-spondin (El-Bitar et al., 2001; Vera et al., 2013, 2015). SCO-spondin knockdown experiments using chick embryos have demonstrated that the protein released into embryonic CSF is required for neurogenesis and regulation of neuroepithelial cell proliferation/neuronal differentiation (Vera et al., 2013). A subsequent study showed that low-density lipoprotein (LDL) and SCO-spondin form a complex and that this interaction is essential in modulating the neuroepithelium differentiation generated by both molecules (Vera et al., 2015). However, *in ovo* inhibition of SCO-spondin using shRNA in the chick embryo has reduced neuronal cell number and increased PCNA-positive cells (Vera et al., 2013). A similar result was obtained by *in vitro* culture with the explanted optic tecta in the SCO explant conditioned medium and SCO-spondin-depleted embryonic CSF (Vera et al., 2015). These results are inconsistent with those from SCO transplantation experiments. Cellular response to the SCO-spondin might be different between embryonic and adult brains, or among species.

## Conclusion

In this review, we highlighted a possible role of the SCO in adult neurogenesis: as a neurogenesis/gliogenesis niche or a neurogenesis/gliogenesis regulatory region through secretory factors. Our knowledge about functions of the SCO in adult neurogenesis remains limited due to lacking SCO specific conditional knockout animals. Only a few studies related to functional disruption of the SCO, especially with SCO-spondin deficient animals, mainly focused on the relationship between the RF formation, CSF flow, and hydrocephalus (Perez-Figares et al., 2001; Sepulveda et al., 2021). For example, immunological blockage of the SCO function was able to induce a hydrocephalus phenotype in adult rats (Vio et al., 2000). Recently, a series of studies using zebrafish showed that a mutation in the SCO-spondin gene (*sspo*) causes a phenotype with an abnormal ventral curvature of the body axis and idiopathic scoliosis (Cantaut-Belarif et al., 2018; Lu et al., 2020; Rose et al., 2020). However, the effect of SCO disruption on adult neurogenesis has not been investigated. There are still enigmas on the SCO function on CSF homeostasis and neurogenesis. Comprehensive transcriptome analysis of genes expressed in the SCO and systematic analysis using SCO-specific conditional knockout animals of the related genes would be essential to elucidate the SCO contribution to adult neurogenesis.

## Author contributions

HI was involved in writing the first manuscript. HI, LC, and NO contributed to the manuscript revision, reviewed, and approved the submitted version of the manuscript. All authors contributed to the article and approved the submitted version.

## Footnotes

1
https://mouse.brain-map.org/experiment/show/112644522

2
https://mouse.brain-map.org/gene/show/13576

3
https://mouse.brain-map.org/
